# Thyroid feedback quantile-based index predicts 90-day all-cause mortality or readmission in hospitalized heart failure patients: a retrospective cohort study

**DOI:** 10.3389/fendo.2026.1857196

**Published:** 2026-07-09

**Authors:** Li Ma, Huijuan Li, Manting Gou, Xingbang Liu, Tong Zhang

**Affiliations:** 1 Department of General Medicine, Heze Hospital Affiliated to Shandong First Medical University, Shandong, China; 2 Department of General Medicine, Shandong Provincial Hospital Heze Branch, Shandong, China; 3 Department of Endocrinology and Metabolism, Tianjin University Central Hospital, Tianjin, China; 4 Department of Cardiology, Heze Hospital Affiliated to Shandong First Medical University, Shandong, China; 5 Department of Cardiology, Heze Municipal Hospital, Shandong, China

**Keywords:** heart failure, mortality, readmission, TFQI, thyroid feedback quantile-based Index

## Abstract

**Objective:**

The Thyroid Feedback Quantile-Based Index (TFQI), a recently developed indicator derived from thyroid stimulating hormone (TSH) and free thyroxine (FT4) levels, has demonstrated prognostic value in metabolic diseases. Nevertheless, its utility in predicting clinical endpoints among heart failure (HF) patients remains unclear. This study aimed to identify the optimal TFQI cutoff for risk stratification and to evaluate its association with adverse outcomes in HF patients.

**Methods:**

A total of 402 HF patients were enrolled in the study. Using the composite outcome of 90-day all-cause mortality or heart failure readmission, the X-tile project determined the optimal TFQI threshold. TFQI’s predictive value was then evaluated using univariate and multivariate Cox regression, restricted cubic spline (RCS) analysis, and Kaplan-Meier curves.

**Results:**

A threshold of 0.10 best stratified high-risk patients (adjusted HR: 1.95, 95% CI: 1.11–3.43, P = 0.019). RCS analysis indicated a linear dose-response (P for non-linearity = 0.677). Kaplan–Meier analysis confirmed significantly worse survival with TFQI > 0.10 vs ≤ 0.10 (Log-rank P = 0.004).

**Conclusions:**

A TFQI exceeding 0.10 is associated with a significantly increased risk of 90-day all-cause mortality or HF readmission in hospitalized HF patients. This study suggests TFQI acts as a promising exploratory biomarker correlated with adverse clinical outcomes in HF. Further large-scale, multicenter and prospective studies are still required to verify its clinical value and confirm its role in risk stratification.

## Introduction

Heart failure (HF), a common and complex clinical syndrome, arises from the heart’s inability to pump sufficient blood to meet bodily demands, driving high morbidity and mortality (1). In the United States, HF affects~6.2 million adults, with prevalence projected to rise amid population ageing and growing cardiovascular risk burdens (2). A major challenge in HF management is the high rate of hospital readmissions, with nearly 25% of patients readmitted within 30 days and 35% within 90 days of discharge (3, 4). Additionally, all-cause mortality within 90 days post-discharge remains alarmingly high, ranging from 12% to 20% among HF patients (5, 6). These statistics underscore the urgent need for effective risk stratification tools to improve patient outcomes.

The pathophysiology of HF involves intricate neurohormonal and inflammatory pathways that contribute to progressive cardiac dysfunction (7). Thyroid hormones play a critical role in maintaining cardiovascular homeostasis, influencing myocardial contractility, cardiac rhythm, and vascular resistance (8). Dysregulation of thyroid hormone levels and tissue sensitivity has been linked to adverse outcomes in HF (9). Notably, the sensitivity of target tissues to thyroid hormones is a key determinant of clinical outcomes in HF patients (10). Traditional assessments of thyroid function, including measurements of free thyroxine (FT4) and triiodothyronine (FT3), provide limited insight into tissue-level hormonal sensitivity. Emerging research demonstrates that subtle shifts in thyroid hormone levels or sensitivity substantially affect HF outcomes (11). The Thyroid Feedback Quantile-Based Index (TFQI), derived from TSH and FT4 levels, has emerged as a promising prognostic marker for evaluating thyroid function in various clinical contexts (12). Unlike conventional thyroid function tests, TFQI captures the dynamic feedback mechanisms regulating thyroid hormone balance, offering a more nuanced understanding of thyroid resistance (11, 13). While TFQI has shown potential in metabolic syndrome and diabetes, particularly in its association with insulin resistance (11), its application in HF prognosis remains underexplored. Despite its potential, the application of TFQI in HF prognosis remains underexplored. Although low FT3 levels have been associated with poor outcomes in HF patients (14), the relationship between TFQI and critical endpoints such as 90-day readmission and all-cause mortality has not been thoroughly investigated.

Therefore, the current study was designed to explore the relationship between TFQI and heart failure outcomes, with a particular emphasis on 90-day HF readmission rates and overall mortality. By elucidating the connection between TFQI and clinical outcomes in heart failure, our objective is to augment existing knowledge and offer insights that may guide the development of more efficacious management strategies for this vulnerable patient group.

## Methods

### Study population

**Figure 1:**
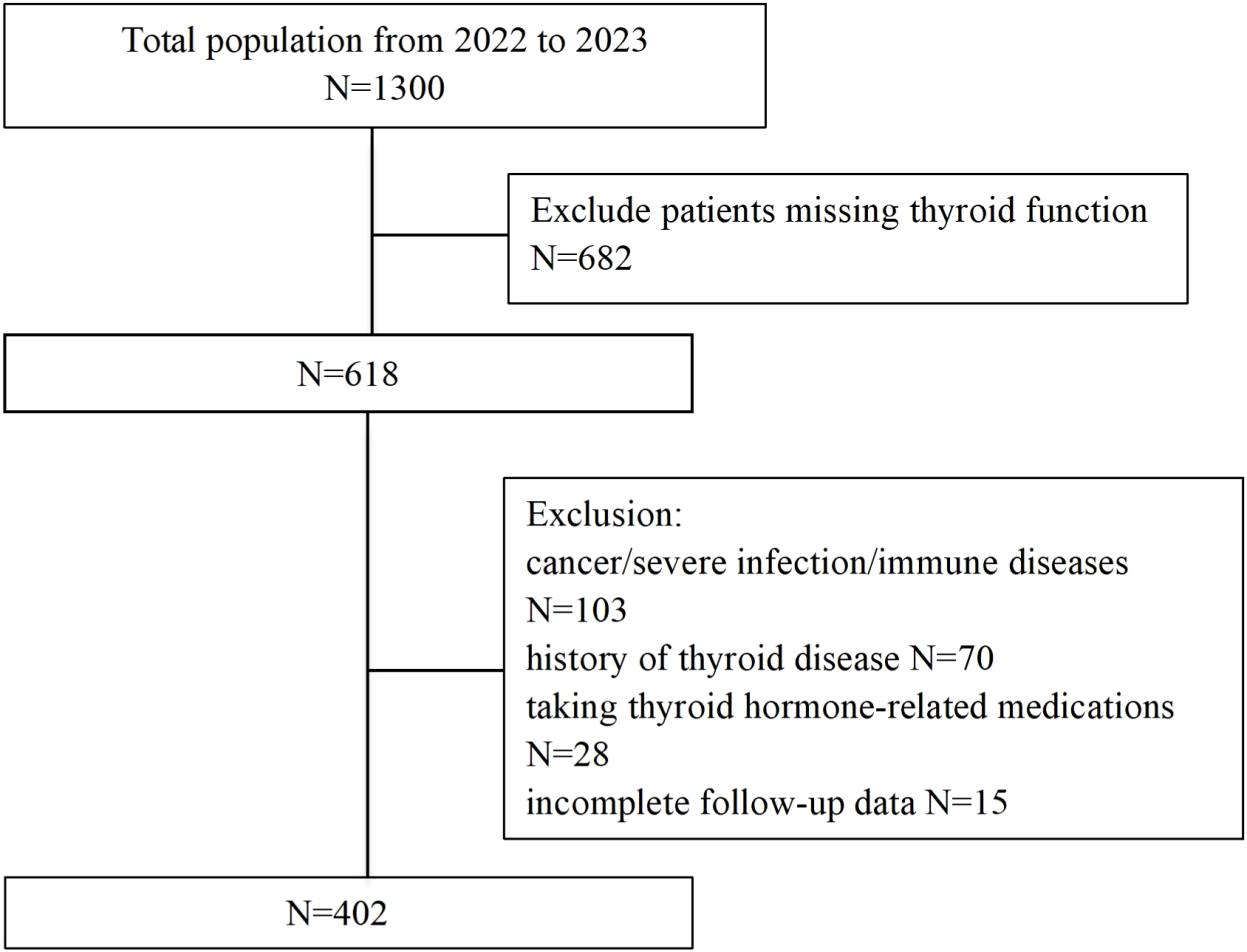
Flow chart of patient selection.

This study encompassed 1, 500 patients with HF admitted to the Cardiology Department of Heze Hospital affiliated to Shandong First Medical University, between January 2022 and June 2023. Eligible participants were aged 18 years or older and met the diagnostic criteria for HF as defined by the Chinese guidelines (2018) (15) and the European Society of Cardiology (2021) (16). Cardiac function was classified according to the New York Heart Association (NYHA) system, which categorizes HF severity into four grades (I–IV) (17). Patients were excluded if they lacked baseline thyroid metrics, a history of thyroid disease or taking medications that could interfere with thyroid function (including amiodarone, lithium, glucocorticoids, dopamine, or any thyroid hormone preparations), cancer, severe infections, or declined follow-up. We enrolled 402 patients in this analysis (**Figure 1**). Ethical approval was obtained from Heze Hospital Ethics Committee (2024-KY001-079) following Helsinki Declaration principles. Written informed consent was secured from all participants.

### Clinical data collection

We abstracted clinical data from electronic medical records and collected venous blood samples within 24 hours of admission. The extracted data included sex, age, body mass index (BMI), smoking and drinking status, heart rate (HR), systolic blood pressure (SBP), NYHA class, HF etiology (hypertension, diabetes mellitus (DM), and coronary heart disease (CHD), as well as medication information (angiotensin-converting enzyme inhibitors (ACEI), angiotensin receptor blocker (ARB), beta-blocker (β), and statins). Left ventricular ejection fraction (LVEF) and left ventricular end-diastolic dimension (LVDD) were measured using the Biplane Simpson echocardiography method (Philips EPIQ 7C). Body mass index (BMI) was calculated and reported as kg/m². Smoking was defined as having smoked ≥100 lifetime cigarettes and currently smoking intermittently or daily (18). Alcohol consumption was classified as regular drinking in the past 12 months. Blood pressure was measured with an electronic blood pressure monitor (Omron U725A).

### Laboratory measurements

Thyroid function markers, including TSH, FT3, and FT4, were measured using direct chemiluminescence on the Cobas 601 analyzer (Roche Diagnostics). Furthermore, serum total cholesterol (TC), triglyceride (TG), low-density lipoprotein cholesterol (LDL-C), high-density lipoprotein cholesterol (HDL-C), fasting plasma glucose (FPG), creatinine (Cr), uric acid (UA), and aspartate aminotransferase (AST) levels were quantified using the automatic biochemical analyzer (Roche Cobas 8000). N-terminal pro-b-type natriuretic peptide (NT-proBNP) was measured by electro-chemiluminescence immunoassays on the analyzer (Automated ECL8000 Immunoassay Analyzer).

### TFQI and optimal threshold estimation

The TFQI was calculated using the following formula: the cumulative distribution function (CDF) of free thyroxine (fT4) minus (1- cdf of TSH) (11). The TFQI scale (-1 to 1) quantifies thyroid hormone sensitivity: negative values correlate with increased sensitivity, positive values with decreased sensitivity. The X-tile plots offer a comprehensive evaluation of diverse methodologies for categorizing populations into low, medium, and high marker expression levels. The X-tile software undertakes a rigorous statistical assessment by initially establishing divisions within a “training set” and subsequently validating these divisions in an independent patient cohort (19).

### Outcome

The co-primary endpoints comprised 1) all-cause mortality and 2) HF readmission within 90 days after discharge. All-cause mortality and HF readmission within 90 days post-discharge were identified through hospital electronic medical records combined with structured telephone follow-up. Only readmissions for HF that occurred at our own hospital were documented. All events were independently adjudicated by two cardiologists, with any disagreements resolved by a third reviewer.

### Statistics analysis

Statistical analyses were performed using SPSS version 25.0 and the R package version 4.2.1. To compare continuous variables, the Mann–Whitney U-test was utilized, while categorical variables were analyzed using the χ2 or Fisher’s exact test. Univariate Cox regression was used to identify significant predictors of adverse outcomes, which were subsequently included in multivariate Cox regression models. The proportional hazards assumption for the final Cox model was tested using Schoenfeld residuals. RCS analysis with knots at the 10th, 50th, and 90th percentiles was employed to evaluate the non-linear relationship between TFQI and adverse outcomes. Kaplan-Meier analysis with log-rank testing evaluated survival differences. P value < 0.05 was considered statistically significant.

## Results

The cohort comprised 402 patients (**Table 1**). Participants had a median age of 73 years (67, 79), and 58% were male (n = 233). Within 90 days, 65 patients (16.2%) experienced either readmission or all-cause mortality. No patients were lost during the 90-day follow-up. Compared to patients without composite endpoint events, those who experienced adverse outcomes exhibited significantly elevated levels of NT-proBNP, TFQI, as well as lower levels of TC (P < 0.05 for all). Additionally, the prevalence of diabetes was higher in the event group, whereas the incidence of coronary heart disease and the proportion of males were lower.

**Table 1:** Baseline characteristics of the study population (n = 402).

Variable	Overall(n=402)	No readmission or death(n=337)	Readmission or death (n=65)	P
Age (years)	73(67, 79)	73 (65, 79)	75(69, 80)	0.129
Gender, n (%)				
Male	233 (58.0)	204 (60.5)	29 (44.6)	0.017
Female	169 (42.0)	133 (39.5)	36 (55.4)	
BMI (kg/m^2^)	23.3 (20.8, 26.0)	23.4 (20.8, 26.0)	22.7 (20.2, 25.7)	0.519
Smoking (%)	108 (26.9)	93 (27.6)	15 (23.1)	0.452
Drinking (%)	53 (13.2)	49 (14.5)	4 (6.2)	0.067
HR (bpm)	86 (72, 100)	86 (72, 101)	88 (63, 99)	0.424
NYHA(III-IV)	359 (89.3)	298 (88.4)	61 (93.8)	0.341
LVEF, %	42 (34, 53)	43 (35, 53)	40 (33, 53)	0.704
LVDD (mm)	56 (49, 64)	57 (49, 63)	56 (48, 64)	0.930
NT-proBNP (pg/ml)	5957 (2831, 10528)	5217 (2579, 9825)	8185 (4361, 12952)	0.005
SBP (mmHg)	130 (114, 148)	130 (113, 148)	132 (115, 150)	0.631
FT4 (ng/dl)	1.48 (1.31, 1.66)	1.47 (1.31, 1.65)	1.51 (1.27, 1.68)	0.726
FT3 (pg/ml)	2.44 (2.06, 2.84)	2.46 (2.10, 2.85)	2.28 (1.90, 2.66)	0.057
TSH (uIU/ml)	3.03 (1.84, 4.83)	2.93 (1.78, 4.64)	3.71 (2.20, 5.46)	0.079
TFQI	0.01 (-0.26, 0.28)	-0.02 (-0.27, 0.25)	0.13 (-0.13, 0.37)	0.031
TC (mmol/L)	3.62 (2.97, 4.29)	3.65 (3.05, 4.32)	3.47 (2.70, 4.03)	0.030
TG (mmol/L)	0.91 (0.72, 1.28)	0.91 (0.72, 1.29)	0.96 (0.72, 1.20)	0.837
LDL (mmol/L)	2.05 (1.58, 2.71)	2.08 (1.63, 2.74)	1.70 (1.36, 2.66)	0.097
HDL (mmol/L)	0.97 (0.80, 1.21)	0.98 (0.80, 1.21)	0.91 (0.78, 1.27)	0.454
FPG (mmol/L)	4.86 (4.29, 5.84)	4.82 (4.24, 5.74)	5.07 (4.35, 6.15)	0.258
Cr (umol/L)	85 (68, 105)	84 (68, 103)	89 (72, 111)	0.220
UA (umol/L)	350 (274, 456)	345 (273, 454)	363 (282, 484)	0.374
AST (U/L)	23 (16, 33)	23 (17, 34)	20 (16, 31)	0.411
Hypertension (%)	208 (51.7)	170 (50.4)	38 (58.5)	0.236
Diabetes (%)	104 (25.9)	80 (23.7)	24 (36.9)	0.026
CHD (%)	195 (48.5)	172 (51.0)	23 (35.4)	0.021
ACEI/ARB (%)	282 (70.1)	239 (70.9)	43 (66.2)	0.442
β-Blocker (%)	308 (76.6)	261 (77.4)	47 (72.3)	0.370
Statin (%)	305 (75.9)	255 (75.7)	50 (76.9)	0.829

BMI, body mass index; HR, Heart rate; NYHA, New York Heart Association; LVEF, Left ventricular ejection fraction; LVDD, left ventricular end-diastolic diameter; NT-proBNP, N-terminal pro-b-type natriuretic peptide; SBP, systolic blood pressure; FT4, free thyroxine; FT3, free triiodothyronine; TSH, thyrotropin; TFQI, thyroid feedback quantile-based index; TC, total cholesterol; TG, triglycerides; LDL, low-density lipoprotein; HDL, high-density lipoprotein; FPG, fasting plasma glucose; Cr, creatinine; UA, uric acid; AST, aspartate aminotransferase; CHD, coronary heart disease. ACEI, angiotensin converting enzyme inhibitor; ARB, angiotensin receptor blocker; β-Blocker, Beta Blocker.

**Figure 2:**
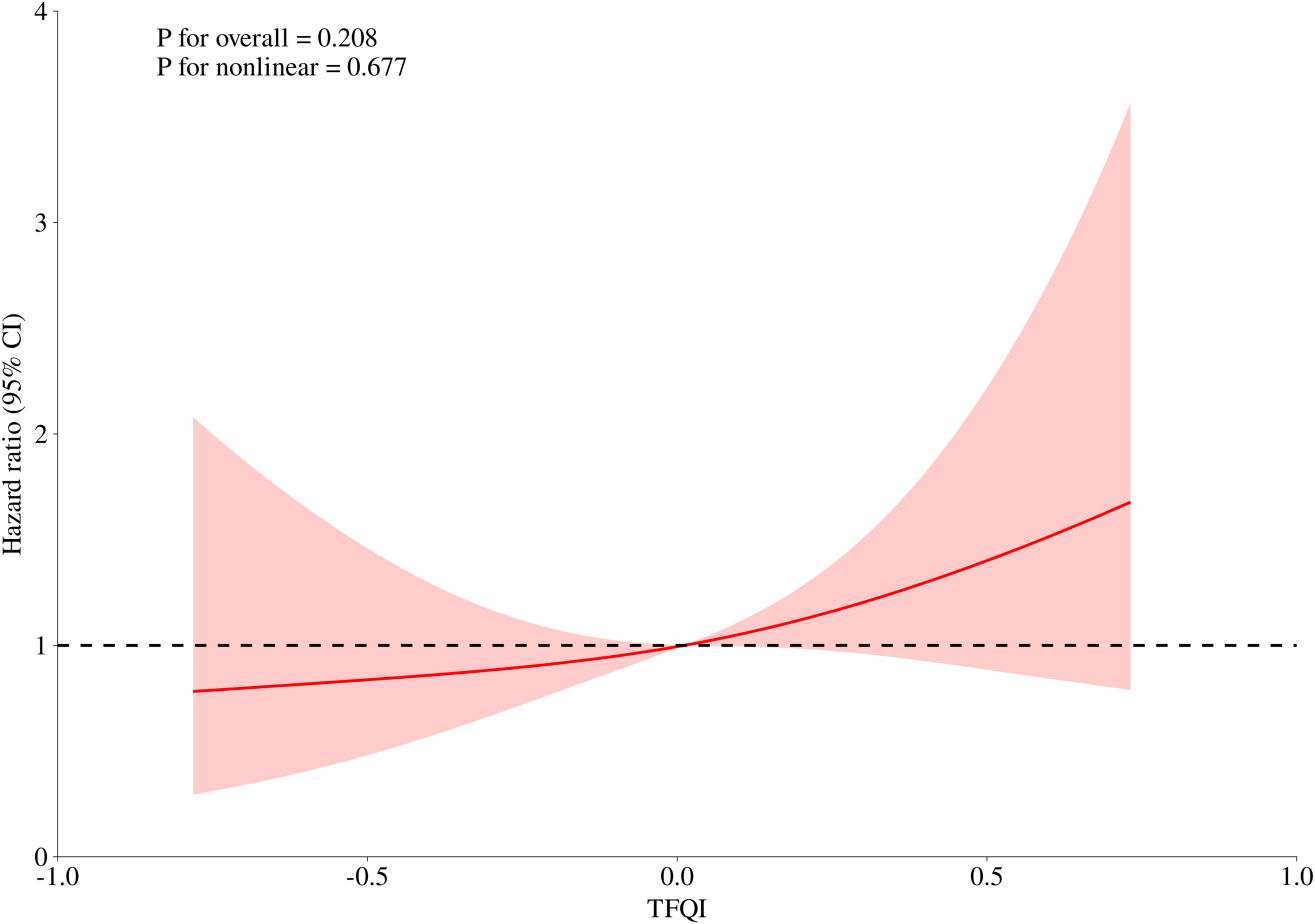
RCS analysis results of TFQI and mortality or readmission using Cox regression model. Adjusted HRs are indicated by a solid line and 95% confidence intervals by red color derived from RCS with 3 knots.

**Figure 3:**
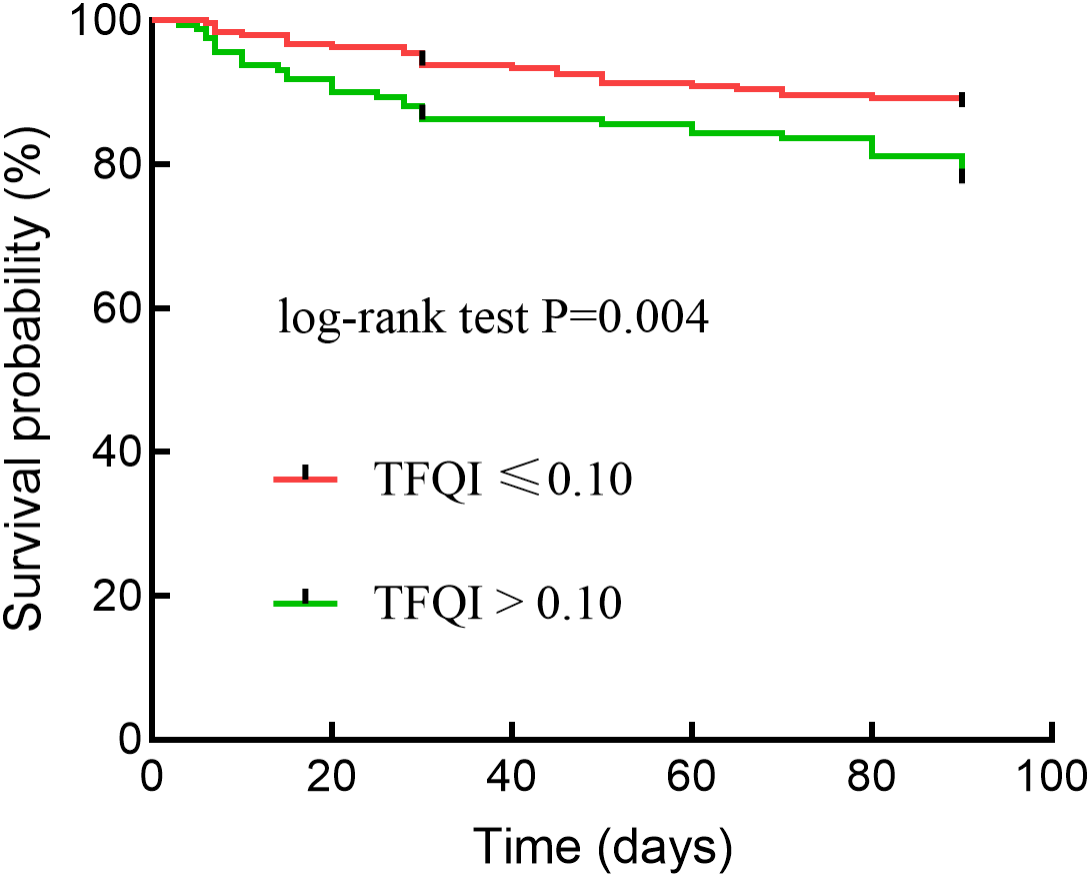
Survival curves in relation to 90-day all-cause mortality and readmission stratified by TFQI value.

The TFQI cutoff value predictive of composite endpoints was established at 0.10 through X-tile analysis. As shown in **Table 2**, patients with TFQI > 0.10 had higher levels of NT-proBNP, FT4, TSH, and UA, as well as a greater incidence of composite endpoints, compared to those with TFQI ≤ 0.10. Traditional risk factors, including demographic variables (age, sex), clinical parameters (BMI, smoking, alcohol consumption, NYHA class, LVEF, LVDD, SBP), laboratory markers (TC, TG, LDL, HDL, Cr, FPG), and medical history (hypertension, diabetes, CHD), were evaluated. Univariate Cox regression analysis identified several significant predictors of 90-day all-cause mortality or readmission, including female sex, log-transformed NT-proBNP, creatinine levels, a history of diabetes mellitus (DM), and TFQI > 0.10 (**Table 3**). According to the Schoenfeld residuals test, the proportional-hazards assumption of the Cox model was met (global test P = 0.34), indicating no violation of the assumption. After adjusting for sex, logNT-proBNP, creatinine, and diabetes in multivariable Cox models, TFQI > 0.10 remained associated with composite outcomes (adjusted HR: 1.89, 95% CI: 1.14–3.12, P = 0.013). RCS analysis revealed a linear positive relationship between TFQI values and the risk of composite adverse outcomes (P for non-linearity = 0.677; **Figure 2**). Kaplan-Meier survival analysis further demonstrated a significant difference in survival between patients with TFQI > 0.10 and those with TFQI ≤ 0.10 (Log-rank test: P = 0.004; **Figure 3**).

**Table 2:** Characteristics according to TFQI (n = 402).

Variables	TFQI ≤ 0.10 (n=242)	TFQI > 0.10 (n=160)	P
Age (years)	74 (67, 80)	73(65, 79)	0.217
Gender, n (%)			
Male	143 (59.1)	90 (56.3)	0.572
Female	99 (40.9)	70 (43.7)	
BMI (kg/m^2^)	23.47 (20.76, 26.27)	23.09 (20.31, 24.97)	0.112
Smoking (%)	68 (28.1)	40 (25.0)	0.493
Drinking (%)	34 (14.0)	19 (11.9)	0.528
HR (bpm)	87 (74, 100)	85 (70, 100)	0.226
NYHA (III-IV), %	216 (89.3)	143 (89.4)	0.970
LVEF, %	42 (33, 54)	43 (35, 52)	0.992
LVDD (mm)	57 (49, 63)	56 (48, 64)	0.991
NT-proBNP (pg/ml)	4915 (2406, 8819)	6927 (3892, 12778)	<0.001
SBP (mmHg)	132 (116, 148)	129 (111, 150)	0.640
FT4 ((ng/dl)	1.36 (1.23, 1.50)	1.63 (1.52, 1.86)	<0.001
FT3 (pg/ml)	2.40 (2.06, 2.83)	2.47 (2.11, 2.84)	0.410
TSH (uIU/ml)	2.18 (1.41, 3.44)	4.44 (3.12, 6.27)	<0.001
TC (mmol/L)	3.67 (3.05, 4.30)	3.54 (2.90, 4.28)	0.304
TG (mmol/L)	0.91 (0.71, 1.28)	0.92 (0.72, 1.27)	0.807
LDL (mmol/L)	2.08 (1.62, 2.74)	1.98 (1.55, 2.69)	0.284
HDL (mmol/L)	1.00 (0.81, 1.25)	0.93 (0.77, 1.20)	0.199
FPG (mmol/L)	4.85 (4.34, 5.76)	4.92 (4.15, 5.96)	0.554
Cr (umol/L)	82 (67, 104)	88 (73, 107)	0.085
UA (umol/L)	333 (262, 452)	368 (284, 480)	0.028
AST (U/L)	22 (16, 34)	23 (17, 32)	0.432
Hypertension (%)	124 (51.2)	79 (49.4)	0.714
Diabetes (%)	60 (24.8)	44 (27.5)	0.544
CHD (%)	125 (51.7)	85 (53.1)	0.771
ACEI/ARB (%)	171 (70.7)	111 (69.4)	0.783
β-Blocker (%)	182 (75.2)	126 (78.8)	0.411
Statin (%)	189 (78.1)	116 (72.5)	0.199
Readmission or death (%)	29 (12.0)	36 (22.5)	0.005

BMI, body mass index; HR, Heart rate; NYHA, New York Heart Association; LVEF, Left ventricular ejection fraction; LVDD, left ventricular end-diastolic diameter; NT-proBNP, N-terminal pro-b-type natriuretic peptide; SBP, systolic blood pressure; FT4, free thyroxine; FT3, free triiodothyronine; TSH, thyrotropin; TC, total cholesterol; TG, triglycerides; LDL, low-density lipoprotein; HDL, high-density lipoprotein; FPG, fasting plasma glucose; Cr, creatinine; UA, uric acid; AST, aspartate aminotransferase; CHD, coronary heart disease. ACEI, angiotensin converting enzyme inhibitor; ARB, angiotensin receptor blocker; β-Blocker, Beta Blocker; TFQI, thyroid feedback quantile-based index.

**Table 3:** Univariate and multivariate cox’s regression analyses for 90-day mortality or readmission.

Variable	Univariate analysis		Multivariate analysis	
	HR(95% CI)	P	HR(95% CI)	P
Gender				
Male	Ref			
Female	1.77 (1.09, 2.89)	0.022	1.71 (1.04, 2.82)	0.036
Age (years)	1.02 (0.99, 1.044)	0.084		
BMI (kg/m2)	1.00 (0.94, 1.04)	0.658		
Smoking	0.80 (0.45, 1.42)	0.445		
Drinking	0.41 (0.15, 1.20)	0.082		
HR (bpm)	0.99 (0.98, 1.01)	0.312		
NYHA				
I-II	Ref			
III-IV	1.88 (0.68, 5.16)	0.223		
LVEF, %	0.99 (0.98, 1.02)	0.842		
LVDD	1.00 (0.98, 1.02)	0.903		
Log-NTproBNP (pg/ml)	2.15 (1.17, 3.95)	0.014	1.45 (0.76, 2.74)	0.258
SBP (mmHg)	1.00 (0.99, 1.01)	0.636		
TC (mmol/L)	0.81 (0.63, 1.05)	0.109		
TG (mmol/L)	0.91 (0.60, 1.38)	0.648		
LDL (mmol/L)	0.83 (0.62, 1.13)	0.236		
HDL (mmol/L)	0.82 (0.37, 1.86)	0.641		
FPG (mmol/L)	1.07 (0.98, 1.17)	0.156		
Cr (umol/L)	1.00 (1.00, 1.01)	0.031	1.00 (1.00, 1.01)	0.062
UA (umol/L)	1.00 (1.00, 1.00)	0.467		
AST (U/L)	1.00 (1.00, 1.01)	0.698		
Hypertension	1.03 (0.64, 1.68)	0.894		
Diabetes	1.75 (1.06, 2.90)	0.029	1.45 (0.86, 2.44)	0.161
CHD	1.30 (0.79, 2.13)	0.296		
ACEI/ARB	0.83 (0.49, 1.38)	0.456		
β-Blocker	0.80 (0.46, 1.34)	0.420		
Statin	1.03 (0.58, 1.83)	0.934		
TFQI				
>0.10	2.003 (1.228, 3.266)	0.005	1.89 (1.14, 3.12)	0.013
≤0.10	Ref			

HR, hazard ratio; BMI, body mass index; HR, Heart rate; NYHA, New York Heart Association; LVEF, left ventricular ejection fraction; LVDD, left ventricular end-diastolic diameter; NT-proBNP, N-terminal pro-b-type natriuretic peptide; SBP, systolic blood pressure; TC, total cholesterol; TG, triglycerides; LDL, low-density lipoprotein; HDL, high-density lipoprotein; FPG, fasting plasma glucose; Cr, creatinine; UA, uric acid; AST, aspartate aminotransferase; CHD, coronary heart disease. ACEI, angiotensin converting enzyme inhibitor; ARB, angiotensin receptor blocker; β-Blocker, Beta Blocker; TFQI, thyroid feedback quantile-based index.

## Discussion

In recent years, the TFQI has emerged as a promising prognostic marker for various diseases. However, its application in HF prognosis remains underexplored, and a definitive threshold for identifying high-risk patients has yet to be established. This study, involving 402 hospitalized HF patients, investigated the association between TFQI and 90-day outcomes, including mortality and readmission. Our key findings indicate that a TFQI threshold of 0.10 effectively identifies patients with significantly higher risk of poor prognosis. Patients with TFQI > 0.10 had a significantly increased risk of 90-day all-cause mortality and readmission compared to those with TFQI ≤ 0.10. Kaplan-Meier survival analysis further confirmed that survival rates were significantly lower in the TFQI > 0.10 group, underscoring the predictive utility of this biomarker. Additionally, RCS analysis revealed a linear positive relationship between TFQI and composite adverse event risk, indicating that higher TFQI values correlate with worse prognosis.

When compared to other established prognostic biomarkers in HF, such as B-type natriuretic peptide (BNP) and NT-proBNP, TFQI offers unique advantages. While BNP and NT-proBNP are widely recognized for their predictive value in hemodynamic stress, they may not fully capture the complex hormonal interactions underlying cardiovascular health (20, 21). In contrast, TFQI integrates thyroid hormone levels (FT4 and TSH) into a single metric, reflecting the balance of thyroid feedback regulation and providing a more comprehensive assessment of thyroid function. This approach may hold particular value for identifying patients with subtle thyroid dysfunction at increased risk of adverse outcomes.

Thyroid hormones play a critical role in cardiovascular homeostasis, influencing gene expression, myocardial contractility, and cardiac output (22). Previous studies have demonstrated that HF patients with diminished thyroid hormone levels face elevated risks of adverse cardiac outcomes, highlighting the importance of early diagnosis and management of thyroid dysfunction (23). Both hypothyroidism and hyperthyroidism have been associated with the initiation and progression of HF (24, 25). Our findings align with these studies, reinforcing the notion that thyroid hormone modulation plays a crucial role in HF outcomes.

The mechanisms by which TFQI influences HF risk can be conceptualized through three potential pathways. First, elevated TFQI may indicate a pro-inflammatory state characterized by higher levels of free thyroxine (FT4), which could activate the sympathetic nervous system, exacerbate HF symptoms, and increase readmission rates (26). Second, increased TFQI may reflect disrupted thyroid hormone regulation, leading to maladaptive responses such as heightened myocardial oxygen demand and worsened HF symptoms (27). Third, thyroid hormones are essential for maintaining energy homeostasis (28). It has been hypothesized that metabolic dysfunction-associated steatotic liver disease (MASLD) may serve as a potential mediator in the relationship between elevated TFQI and poor HF outcomes, based on previous literature (29, 30). However, we did not collect liver imaging or fibrosis data, and this mechanistic speculation is not directly tested in our study; it is presented only as a hypothesis for future investigation.

Our study also highlights significant gender disparities in HF outcomes, with women facing a higher risk of 90-day readmission and all-cause mortality compared to men. This finding is consistent with prior research indicating that women often exhibit more severe clinical manifestations and poorer outcomes in HF (31, 32). Potential explanations for these disparities include hormonal fluctuations, particularly the cardioprotective effects of estrogen, which influence endothelial function and inflammatory responses (33). However, postmenopausal women experience a decline in estrogen levels, potentially elevating their risk of cardiovascular events. Additionally, women are more likely to develop heart failure with preserved ejection fraction (HFpEF), which has distinct pathophysiological characteristics and is associated with poorer outcomes compared to heart failure with reduced ejection fraction (HFrEF) (34). Gender-specific variations in comorbidities and treatment responses may also influence these outcomes. For example, women with diabetes have a significantly higher risk of cardiovascular events and all-cause mortality than men (35, 36). These observations underscore the need for gender-specific strategies to optimize HF management.

To our knowledge, this exploratory study represents the first attempt to identify an optimal TFQI threshold for stratifying mortality and readmission risk in hospitalized HF patients, but the findings should be interpreted with caution. Previous research has established similar associations in other clinical contexts. For instance, elevated TFQI has been linked to metabolic dysfunction-associated fatty liver disease in euthyroid individuals (37) and an increased risk of coronary artery disease in prospective cohort studies (38, 39). In the United States, TFQI has been associated with a higher prevalence of diabetes among euthyroid adults (40). Furthermore, studies in Chinese populations have identified correlations between higher TFQI levels and abdominal obesity, hypertriglyceridemia, and hypertension (41). Additionally, another cross-sectional study found significant relationships between TFQI and conditions such as diabetes and hypertension (42, 43). These findings suggest that even subtle variations in thyroid hormone levels can have significant implications for patient outcomes.

The identification of TFQI as a prognostic marker in HF, particularly with a cutoff value of 0.10, has important clinical implications. Routine evaluation of thyroid function should be integrated into the management of HF patients, given the ease of measuring thyroid hormone levels and the potential impact on outcomes. However, as no external validation or independent validation has been conducted, this threshold should be regarded as an exploratory finding.

Despite these notable findings, our study has several limitations. First, its observational design prevents causal inferences. Second, the limited number of events (n=65) restricted the complexity of multivariable adjustment and precluded separate analyses for mortality and readmission; the composite endpoint is heterogeneous. Third, the TFQI cutoff was derived in the same cohort without internal or external validation, introducing a risk of optimistic bias. Fouth, we did not systematically exclude subclinical thyroid dysfunction, and we lacked data on HF phenotypes. Given these limitations, our findings should be considered exploratory and hypothesis-generating. Larger-scale, ideally multicenter, studies with prospective design are needed to validate the clinical utility of TFQI.

In summary, our study highlights the TFQI associated with a significantly increased risk of 90-day rehospitalization and mortality in HF patients. A linear association was observed between TFQI and adverse endpoints. As an exploratory biomarker, TFQI shows preliminary prognostic value for HF. Additional well-designed studies are needed to clarify its clinical role and explore its application in clinical practice.

## Data Availability

The original contributions presented in the study are included in the article/supplementary material. Further inquiries can be directed to the corresponding author.
